# Transportation Matters: A Health Impact Assessment in Rural New Mexico

**DOI:** 10.3390/ijerph14060629

**Published:** 2017-06-13

**Authors:** Michelle Del Rio, William L. Hargrove, Joe Tomaka, Marcelo Korc

**Affiliations:** 1 Center for Environmental Resource Management, University of Texas at El Paso, 500 W. University Ave., El Paso, TX 79968, USA; wlhargrove@utep.edu; 2 College of Health and Social Services, New Mexico State University, P.O. Box 30001, Las Cruces, NM 88003, USA; tomaka@ad.nmsu.edu; 3 Pan American Health Organization/World Health Organization, 525 23rd St., Washington, DC 20037, USA; korcmarc@paho.org

**Keywords:** Health Impact Assessment, public transportation, rural health

## Abstract

This Health Impact Assessment (HIA) informed the decision of expanding public transportation services to rural, low income communities of southern Doña Ana County, New Mexico on the U.S./Mexico border. The HIA focused on impacts of access to health care services, education, and economic development opportunities. Qualitative and quantitative data were collected from surveys of community members, key informant interviews, a focus group with community health workers, and passenger surveys during an initial introduction of the transit system. Results from the survey showed that a high percentage of respondents would use the bus system to access the following: (1) 84% for health services; (2) 83% for formal and informal education opportunities; and (3) 81% for economic opportunities. Results from interviews and the focus group supported the benefits of access to services but many were concerned with the high costs of providing bus service in a rural area. We conclude that implementing the bus system would have major impacts on resident’s health through improved access to: (1) health services, and fresh foods, especially for older adults; (2) education opportunities, such as community colleges, universities, and adult learning, especially for young adults; and (3) economic opportunities, especially jobs, job training, and consumer goods and services. We highlight the challenges associated with public transportation in rural areas where there are: (1) long distances to travel; (2) difficulties in scheduling to meet all needs; and (3) poor road and walking conditions for bus stops. The results are applicable to low income and fairly disconnected rural areas, where access to health, education, and economic opportunities are limited.

## 1. Introduction

There is wide recognition that the built environment plays a major role in determining health behaviors and outcomes at the individual and community level. There is growing recognition by many planning, development, and health organizations, such as the Pan American Health Organization/World Health Organization (PAHO/WHO), U.S. Environmental Protection Agency (USEPA), U.S. Centers for Diseases Control and Prevention (USCDC), and U.S. Department of Transportation (USDOT), that public transportation can play a significant role and that public health outcomes are influenced by multiple modes of transportation for different types of users of public transportation services [1,2].

Health Impact Assessment (HIA) has emerged as a beneficial tool for assessing direct and indirect public health impacts of infrastructure improvement such as public transportation. However, all of the published HIAs on public transportation have been conducted in urban areas and, for the most part, have focused around proposals of expanding or improving an already existing service. Dannenburg et al. [3] recently reviewed 73 transportation-related HIAs conducted in the U.S. However, all appear to have been done in urban or peri-urban settings; none were focused on transportation in rural areas. These and other studies have shown that access to health services can be negatively impacted by distance to travel, physical impairment and disabilities, lack of a personal mode of transportation, and/or lack of a driver’s license [4,5,6]. Additionally, level of education has been linked to public health and social and economic development [7,8]; lack of transportation can inhibit access to education. In addition, research has shown improved air quality with greater reliance on public transportation by reducing the numbers of individual vehicles on the road [9,10]. All of these factors can impact public health and all can be influenced by public transportation.

In our review of the literature, we found no HIA that focused on public transportation in rural areas. We conducted an HIA in rural southern Doña Ana County, New Mexico (NM), near the U.S./Mexico border (<65 km). This HIA is unique in that it focused on a decision to extend public transit service from the city of Las Cruces to several rural towns/*colonias* in southern NM, including Chaparral, Anthony, Santa Teresa, Sunland Park, and several others. Planning and implementing public transportation in rural areas is challenging as there are no existing public transportation services, the travel distances are relatively great (30–60 km one way), and it is difficult to meet all residents’ needs and local stakeholder’s interests. Additionally, these primarily low resource communities with high rates of poverty are also challenged by very limited social services that are locally available. Conducting an HIA under these circumstances is also difficult as the outcomes and impacts are more difficult to predict.

In spite of these challenges, public transportation could have large direct and indirect benefits for such communities. Direct benefits could include improved access to health care, while indirect benefits could include improved access to a range of goods and services, such as fresh fruits and vegetables, job training, educational opportunities, recreational activities, and others. The primary goal of our HIA was to assess these direct and indirect impacts, and to inform the decision of the South Central Regional Transit District (SCRTD) of New Mexico and Doña Ana County to extend bus service to rural southern Doña Ana County. The SCRTD first proposed rural services in 2014, and piloted a service that operated from June to November of that year (before our HIA). In that same year, the SCRTD proposed a county gross receipts tax increase on the November ballot to make the service more permanent and to expand services. The proposal failed, and services were terminated due to lack of funding. The SCRTD Board re-started a pilot program using some temporary funding from county and state government with intent to put the referendum on the ballot again at a future date to sustain financial support. We planned our HIA to inform decisions surrounding maintenance of the pilot program, putting the referendum on the ballot again, routes for the new transit system, and the operation and maintenance of the systems. Thus, our goal was to assess direct and indirect impacts of public transportation in rural areas of southern Doña Ana County, with particular focus on access to: (1) health services; (2) fresh fruits and vegetables (because they contribute to a healthy diet); (3) education (both formal and informal); and (4) jobs and other economic activities.

We present this HIA as a case study of the impacts of public transportation in rural areas in the U.S./Mexico border region. It is unique because: (1) it is one of the first HIAs conducted on public transportation in rural areas; and (2) it was conducted in the U.S./Mexico border region in an area with many small communities of predominantly Hispanic populations, a high poverty rate, and very limited locally-available support services. The bus service represented the first and only form of public transportation option for the residents, many of whom are elderly and cannot drive, have only one or no vehicle for family use, and have limited access to basic health, government, and social services. We share lessons learned in conducting this HIA in order to inform the practice of HIA in resource-poor rural communities, which should be applicable to similar areas in the U.S. and developing countries around the world.

## 2. Materials and Methods

### 2.1. Case Study Description

**Figure 1:**
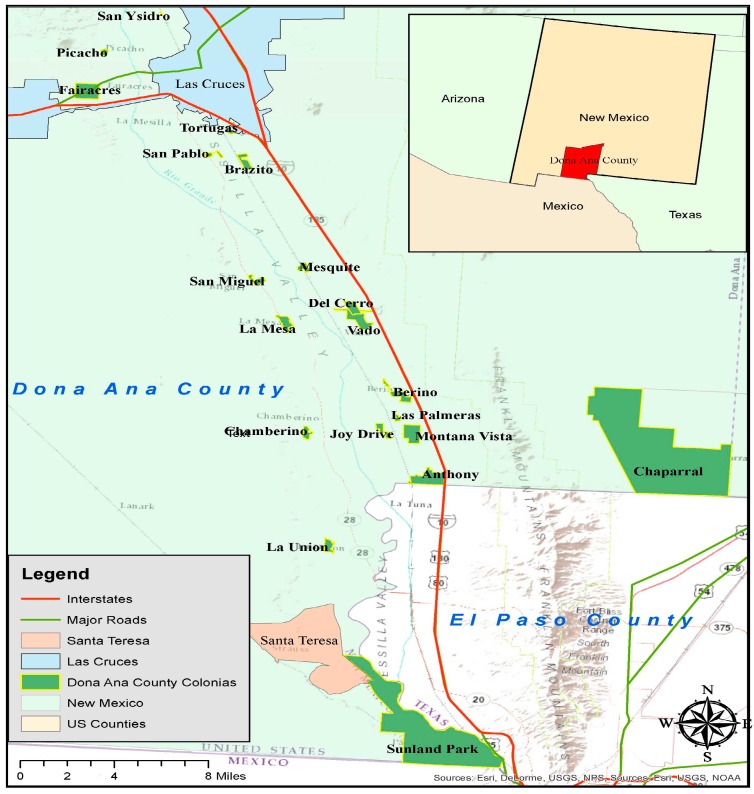
Map of the study area.

Our assessment focused on direct and indirect social determinants of health in relation to the proposed public transportation project. The geographic focus was the area of Doña Ana County located south of Las Cruces, NM (map of study area, Figure 1). The demographics of the study area and of our sample population are presented in Table 1 and discussed in the Results Section.

This area can be characterized as 18 small rural communities between Las Cruces, NM and El Paso, TX (Figure 1). The distance from north to south is about 40 m and the width is about 10 m, for a total area of 400 m^2^. A challenge for transportation is the relatively long distances between communities and the relatively long distance to either Las Cruces or El Paso, where most of the resources that residents in the rural communities need/want to access are located. These needed resources include health services, public community spaces, government offices, educational opportunities, convenience stores, and grocery stores. There is a dearth of these services located in the rural area. There is one family health clinic, La Clinica de la Familia, located near La Union, and there are a few convenience stores in the study area, but otherwise, most of the essential services are concentrated in Las Cruces and near El Paso, TX. El Paso is outside the study area and was not directly served by the SCRTD bus system at the time of our study because it did not have an interstate operating license. Of particular interest to the residents of the study area are several resources in El Paso, including University Medical Center, William Beaumont Army Hospital, major pharmacies near the bus transfer stations, and clinics that offer primary care, dental, or behavioral, mental, and social services to U.S. veterans.

For our assessment, we focused on direct health impacts related primarily to access to health care and fresh fruits and vegetables (because of their impact on a healthy diet, as well as other conditions conducive to safety (such as road accidents) and healthy living (such as walking to bus stops). We collaborated with a local chapter of the Empowerment Congress in Doña Ana County, modeled after a group in Los Angeles, CA. They had conducted a survey among its members to assess the need for and benefits of public transportation in the area. Their results provided some insight into the important indirect impacts to address in our HIA, including: (1) access to formal education by young people who want to attend university or community college; (2) access to informal education such as workforce development training, English classes, citizenship classes, and others; and (3) access to better jobs and economic activities like shopping. Additional issues that we addressed included environmental and larger scale economic impacts related to economic development and the potential for small business development. We focused on these impacts because these conditions are recognized as social determinants of health that optimize health, functionality, and quality of life. We identified the following vulnerable populations to which we gave due consideration in designing our methodology: (1) the elderly, many of whom cannot drive; (2) people with chronic health conditions that require regular treatment; and (3) young adults who seek educational opportunities and/or job training but who do not have a personal vehicle.

### 2.2. Methodology

#### 2.2.1. Health Impact Assessment Methodology

Our methodology incorporated the minimum elements and practice standards for health impact assessment recommended by the North American HIA Practice Standards Working Group [11]. Our HIA followed the six recommended steps of Screening Scoping, Assessment, Recommendations, Reporting, and Monitoring [11,12,13]. We conducted the HIA over a period of 2 years, November 2014 to October 2016, with most of the data collection and survey work conducted over the period of January 2015 to May 2016. The key research questions that guided our assessment work were derived from our Scoping phase. Due to space limitations, we are focusing on the Assessment step, our findings, and the relevance of our work to the practice of HIA. In the following sections, we provide more detail on our methodology for conducting the key informant interviews, community survey, and focus groups. All protocols were approved by the Institutional Review Board for Human Subjects Research at the University of Texas at El Paso (#637598-7). All subjects gave their informed consent for inclusion before they participated in interviews, focus groups, or surveys. We obtained parental consent for individuals who were at least 15 years old but less than 18 years old. We did not survey anyone less than 15 years old.

#### 2.2.2. Community Survey

For the community survey, we developed and administered a 21-question survey (in English or Spanish) for community members. The survey focused on health, education, and economics and was aimed at identifying what kinds of services residents would like to access. We used a convenience sample approach by going to community health clinics, Doña Ana Community College, community centers, senior centers, church parishes, youth farms, and farmers markets and requesting participation in the survey from whomever we encountered. We did not ask individuals to complete the survey if they did not live in the study area. We collected a total of 1054 surveys from 21 different communities in the study area. Some individuals declined to take the survey because they said that they owned a car and were not interested in public transportation. Thus, the surveys that we collected were biased towards individuals who were at least open to the use of public transportation, if not for themselves, for a family member or friend.

#### 2.2.3. Bus Ridership Survey

A bus ridership survey was conducted during the second pilot program. We surveyed bus passengers who rode the bus from their rural community to primarily Las Cruces or Anthony or Sunland Park, NM. The passenger survey consisted of twelve questions asking rural users of SCRTD public transportation their intended destinations, travel time, physical activity gained from walking and cycling to bus stops, perceptions about the bus service, and personal information such as age, gender, and community of residence. Questions were mostly multiple choice on a Likert scale but included open-ended questions. The survey required 5–10 min to complete, including obtaining consent. Surveys were collected over a time period of two weeks in May 2016. For two of the four lines of service, survey administrators rode the buses for two complete round trips to collect surveys from riders. For the other two lines, survey administrators waited at the transfer stations in Anthony and Las Cruces, and interviewed riders as they waited for their transfer bus. We collected 33 survey responses from a total of about 100 passengers/week at the time.

#### 2.2.4. Key Informant Interviews

We interviewed a total of 44 key informants. Key informants were identified through recommendations from local leaders, members of the Empowerment Congress, and key stakeholders. Key informants represented professionals from a range of sectors including health, education, business or economic development, social services, and environment. We used a standard list of 14 open-ended questions to interview each individual. The questions focused on the impacts of public transportation with respect to health, education, economy, workforce development, and environment. Key informants were also asked about their concerns and recommendations for the bus service. Each interview required about 45 min. The interviews were audio recorded, transcribed, and analyzed for qualitative codes. The results were organized into key themes, subthemes, and potential magnitude of impacts. Analysis by the research team and group discussion was conducted to characterize impacts and create a rubric to quantify themes. Main themes included health, education, economy, environment, safety, and infrastructure.

#### 2.2.5. Focus Group

We conducted one focus group comprised of 13 *promotoras*, paraprofessional community health workers who work in the area. The focus group centered on health conditions in the communities and potential benefits of public transportation.

## 3. Results

### 3.1. Demographic Characteristics of Southern Doña Ana County, NM and the Survey Respondents

The demographics of survey respondents are summarized in Table 1, compared to some statistics for Doña Ana County as a whole. A key statistic from this table is that 65% of our survey respondents had household incomes below $20,000/year compared to a median income in Doña Ana County as a whole of $38,426. Clearly, our survey respondents represent a more impoverished segment of the county’s population. Using information from the U.S. Census [14], we also tried to characterize the population in the project study area as a whole. There are almost 60,000 people living in the project area in a total of 20 small communities, 18 of which are unincorporated. Sunland Park and Chaparral are the two largest communities, with about 28,000 total in those two. Households in this area are mostly young families with a median income of about $24,000/year and with about 40% of the total population.

About 40% of the households live below the poverty line. Most households have only one car if any. The study area has a relatively young population; 40% of the population is under 19 years of age. By comparison, our survey sample was comprised of about 31% young adults, and 65% of the households had annual incomes of less than $20,000/year. We did not survey any young people less than 15 years old; so this segment of the population was not included in the survey, though included in the census.

### 3.2. Community Survey

**Figure 2:**
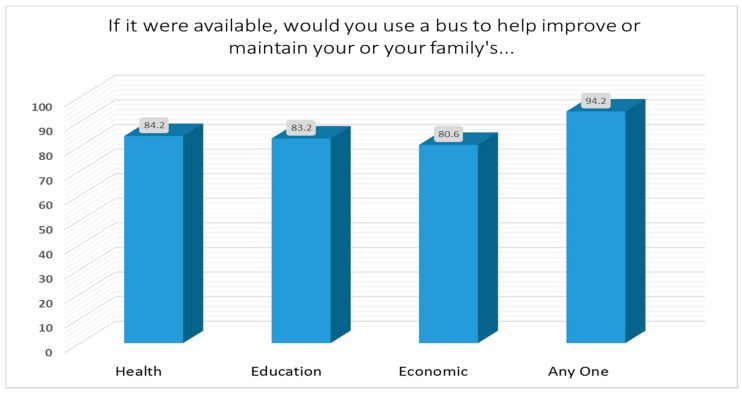
Percentage of respondents who would use the bus service.

For the 1054 respondents to our survey, when asked “if bus service were available, would you or someone in your family use the bus service to improve your…” health, education, or economic situation, an overwhelming majority (>80%) replied that they would (Figure 2). The preferred destinations of respondents were:

- Las Cruces 66% of respondents
- Anthony 42%
- Sunland Park 24%
- Other 19%

The top five reasons for using the bus service for each general category of use are presented in Table 2. Accessing health care was a major reason for using the bus service among older adults. We did not ask about willingness to pay for the bus transit service to access health, education, and economic related activities in the community survey, nor was it mentioned by key informants whom we interviewed.

Accessing education, and especially attending Doña Ana County Community College, was a major reason for using the bus service for young adults. A significant number of young adults (72%) would use public transportation to access job training. In fact, job related opportunities were a much higher preference among young adults than other demographic groups. Seventy-two percent of young adults would use the bus service to access a better job. The high percentage of young adults who would use the bus service to attend community college or university was an unexpected result. We did not anticipate the relatively large number of college-age young people living in the area and still living at home with their families without a car or means to attend college. Public transportation could afford much more opportunity to attend college for this population and lead to a better educated and trained workforce in this economically depressed region.

**Figure 3:**
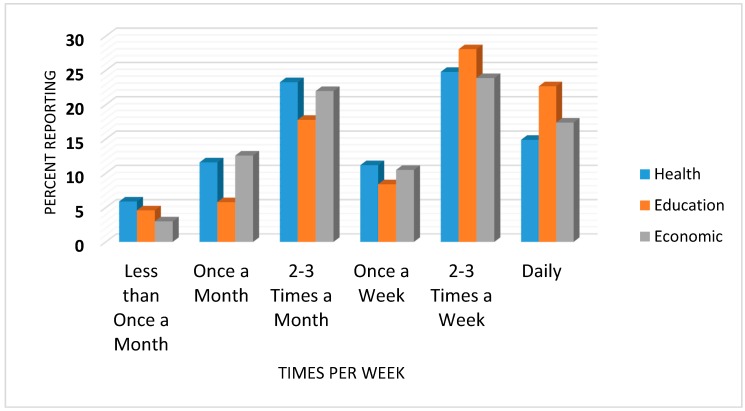
Frequency with which survey respondents would ride the bus for different purposes.

Participants were also asked how often they would use the bus service to access health, education, or economic related activities. Results are presented in Figure 3. For health, the most popular response (24.8%) was “2–3 times a week”, followed by “2–3 times a month” (23.3%). For education and economic activities, the most popular response was also “2–3 times a week” (28.1 and 23.9%, respectively).

### 3.3. Bus Ridership Survey

Table 3 presents the rider characteristics of those who completed the survey. Unfortunately, the number of bus passengers remained small over the summer months in 2016 with ridership ranging from 100 to 150 passengers per week. However, many of these passengers are the same individuals riding multiple days per week. Thus, we surveyed only 33 individuals; we did not complete the survey if respondents said that they had already answered the survey on a previous day or time. The majority of riders were mature adults ranging in age from 45 to 64 (55%). Their main means of transportation was the bus, and they rode most commonly two–five days per week.

**Figure 4:**
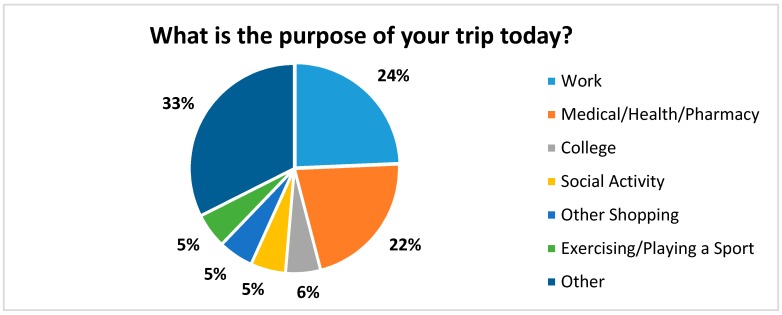
The main purpose for riding the bus.

The main purpose for riding the bus is presented in Figure 4. The most common responses were: other (33%), work (24%), and health care/pharmacy (22%). Some of the reasons associated with “other” included going to a meeting, a Senior Center for a meal, court appearances, visiting El Paso, filing a police report, visiting the casino, grocery shopping, a religious activity, recreation, seeking employment, and job training.

**Figure 5:**
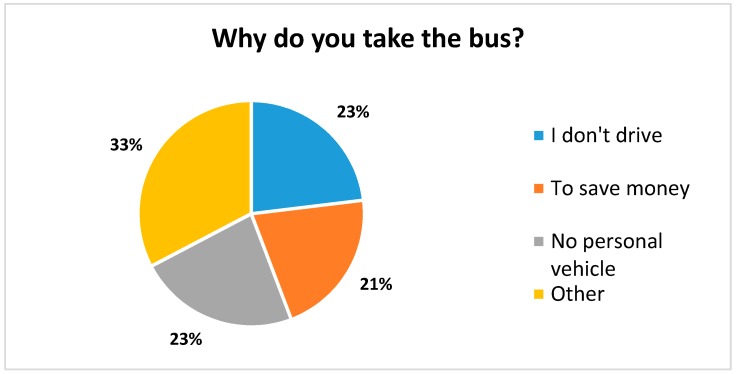
Reasons for taking the bus.

When asked “why did you take the bus?”, the majority of respondents replied either “I don’t drive”, “I don’t have a car”, or “to save money”. The remainder listed other reasons ranging from owning an unreliable car or a shared car, to socializing and being more environmentally friendly (Figure 5).

**Figure 6:**
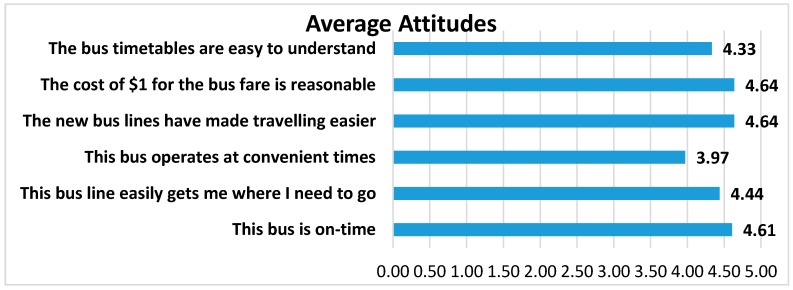
Perceptions about the bus service.

**Figure 7:**
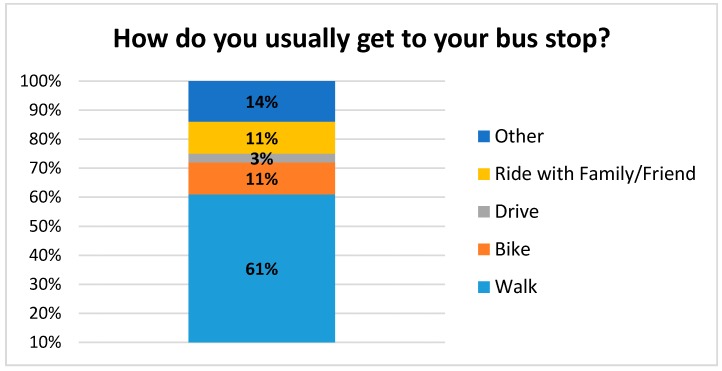
How do you get to your bus stop?

**Figure 8:**
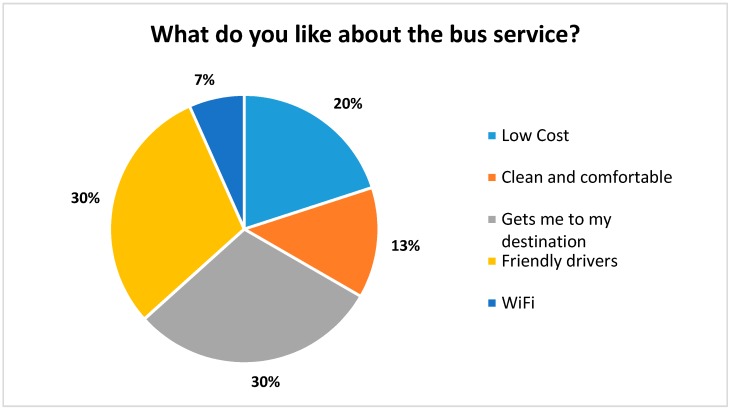
What do you like about the bus service?

**Figure 9:**
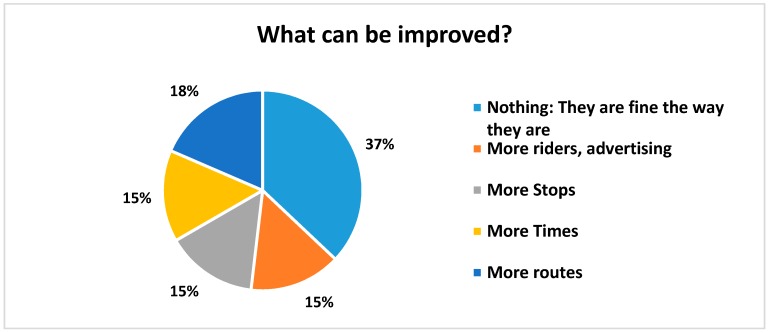
What can be improved about the bus service?

Riders were asked to rate a number of attributes of the bus system on a scale of 1–5 with 5 being the best and 1 being the poorest (Figure 6). Riders were generally very pleased with the overall timeliness, cost, ease of reading the timetables, effectiveness, and efficiency. Regarding the cost of riding the bus and customers’ willingness to pay, we asked if “the cost of $1 for the bus fare is reasonable”, which was the fare at the time. Respondents rated their answer in a Likert-type scale of agreement to disagreement; the average response was 4.64 out of a 5 point scale, with 5 being “strongly agree”. Riders did indicate that the schedules could be more convenient (rating of 3.97 on “the bus operates at convenient times”. The majority of riders walk to their bus stop (61%) and travel an average of 0.78 m in about 20 min (Figure 7). When asked, “what do you like about the bus service?”, the most common answers included the friendly drivers, the low cost, and “Gets me where I am going” (Figure 8). When asked what could be improved, the most common answer was “nothing”, but also several wanted more routes, more times, and more riders/advertising (Figure 9).

### 3.4. Results from Key Informants and Focus Group

The key themes for impacts of public transportation that were identified by key informants are presented in Table 4. These are listed in order of priority. Some additional themes from key informants and the focus group with *promotoras* included safety (especially highway safety but also pedestrian safety), environment (improved air quality), and infrastructure. Highway safety could be improved by reducing substandard vehicles on the roads that do not have good brakes or brake lights, that frequently break down, and create a lot of emissions, and operators who are not insured. Improved air quality could be achieved by decreasing emissions and dust. It was recognized that mass transit could mitigate the risk for non-attainment of air quality for the area of southern Doña Ana and City of Las Cruces. With respect to infrastructure, many said that public transit would improve livability in rural communities, and because of the socialization from improved mobility that rural communities could become more vibrant. In addition, several key informants said that public transit could reduce costs and needs associated to maintain roads and expand highways and streets to accommodate growing traffic.

Some additional themes from key informants and the focus group with *promotoras* included safety (especially highway safety but also pedestrian safety), environment (improved air quality), and infrastructure. Highway safety could be improved by reducing substandard vehicles on the roads that do not have good brakes or brake lights, frequently break down, and create many emissions, as well as operators who are not insured. Improved air quality could be achieved by decreasing emissions and dust. It was recognized that mass transit could mitigate the risk for non-attainment of air quality for the area of southern Doña Ana and City of Las Cruces. With respect to infrastructure, many said that public transit would improve livability in rural communities, and because of the socialization from improved mobility that rural communities could become more vibrant. In addition, several key informants said that public transit could reduce costs and needs associated to maintain roads and expand highways and streets to accommodate growing traffic.

A few negative comments were received and can be summarized as follows:

1. Transportation is not the limiting factor to accessing health services; therefore, there will be limited benefit of providing public transportation to health.
2. Transportation is not the limiting factor to obtaining better jobs; therefore, there will be limited benefit of providing public transportation to economics.
3. Buses will create more congestion.

### 3.5. Impacts of Public Transportation

#### 3.5.1. Summary of Impacts

We identify predicted impacts below for each scoping category of our assessment: (1) access to health services and healthy conditions; (2) access to education; and (3) access to jobs and economic activity/development.

Access to health services and healthy conditions: Both direct and indirect predicted impacts were identified. With respect to direct impacts, the most significant predicted impacts related to improved preventive, short- and long-term care due to improved access to medical services; improved access to pharmaceuticals; and improved access to health-related goods and services. The most impacted populations will be seniors who have greater demand for health care goods and services and less mobility due to either a lack of a personal vehicle or inability to drive. Health service providers in the area informed us that missing appointments was a major problem for residents from the study area. Seeing medical specialists in the Las Cruces area is a particular challenge because of the distance. Indirect impacts include more physical activity to walk or bike to the bus stop and less pollution from the number of cars on the road. Improved road safety is also an important indirect impact. For young people, reducing risky behaviors, such as substance abuse, is a significant indirect impact.

Access to education: Access to educational opportunities is a significant indirect health impact for young adults. Many young people in the area live with their parents. They cannot afford their own car, and are dependent on their parents’ car or a friend. Access to the NMSU Community College campuses is especially important. Improved access to formal education could also have a major impact on the economic development of the region since a college education would improve job opportunities for young people from this area. Improved access to adult learning, especially life skills training and English language training, is also an important outcome for older adults. Parents of school children could also benefit from public transportation that would improve access to the public schools to meet with teachers or attend school related meetings (like School Board meetings).

Access to jobs/economic activity/economic impacts: The most significant positive impacts related to the economy are improved access to jobs and job training. This would be an indirect health impact for families living in the area. The improved access to shopping should also bring economic opportunity to retail businesses in Las Cruces. Significant negative impacts include the cost of the system, which must be borne mostly by taxpayers in Doña Ana County. A lesser negative impact is the potential wear and tear on the rural roads stemming from the buses.

#### 3.5.2. Impacts of Transportation on Access to Health Services and Its Economic Benefits

Lack of transportation can be a major barrier to access to health services and therefore to the prevention of diseases and premature mortality. This is a particular challenge for residents of rural Doña Ana County. We identified and analyzed several challenges with regards to access to health services, impacts of transportation, and potential costs of lack of transportation. There are different methods available to model health costs, including cost-benefit and cost-effectiveness studies, methods approached by other HIA practitioners [16,17], methods adopted by leading health and environmental governmental agencies like the USCDC, USEPA, and international organizations such as the WHO, and transportation and planning agencies (USDOT; U.S. Department of Health and Human Services). However, we did not attempt to monetize health care costs as we did not have access to the data that would allow us to do such an analysis.

Key health conditions for which access to more specialized health care is needed and which are common in our study include (in no certain order):

- All cancers
- Nutritional anemias
- Diabetes mellitus
- Malnutrition
- Suicide
- Diseases of the circulatory system
- Diseases of the respiratory system
- Traffic related injuries
- Alcohol consumption/abuse
- Drug overdose
- Mental, behavioral and neurodevelopmental disorders

In Table 5, we estimate the prevalence of these health conditions in the study area of our HIA from state of New Mexico statistics and the population of our study area or the percentage of the population of Doña Ana County that is represented by our study area (15%). The most prevalent chronic conditions include hypertension, diabetes, depression, and asthma. The consequences of not treating these conditions can lead to more serious respiratory, cardiovascular, and mental conditions. Work absenteeism is also particularly high for these chronic conditions. It is hard to estimate the economic value of treatment of these chronic conditions before they become more serious life-threatening illnesses, but it is clear that access to health services is a crucial determinant in the prevention of more serious illnesses. For our study area, the average travel distance for specialized health services is about 20 m (to either Las Cruces, NM or to El Paso, TX). Reliable transportation becomes a major determinant in accessing needed facilities.

#### 3.5.3. Economic Benefit of Preventive Health Services

The economic benefit of improved access to health services is very difficult to quantify for a rural area like southern Doña Ana County. Preventive health services can reduce the significant economic burden of disease in addition to improving the length and quality of people’s lives. For example, regular preventive care can lead to early detection of cancer, reducing the cost of treatment and premature mortality. Treatment, lost productivity, and health care costs are significant burdens to the economy, families, and businesses. Prevention policies and programs often are cost-effective, reduce health care costs, and improve productivity. Accepting that public transportation would improve access to preventive health services, below we provide several examples of economic benefits that could be at least partially realized by improving access to preventive health services through public transportation. These examples are from the National Prevention Strategy of the U.S. Department of Health and Human Services [18]; there are no such estimates available specifically for the region or for the state of New Mexico. However, assuming that these national estimates are applicable to the region, it is clear that prevention is cost effective.

- A proven program that prevents diabetes may save costs within three years. One of every five U.S. health care dollars is spent on caring for people with diagnosed diabetes. People who increased physical activity (2.5 h/week) and had 5–7% weight loss reduced their risk of developing type 2 diabetes by 58% regardless of race, ethnicity, or gender.
- A 5% reduction in the prevalence of hypertension would save $25 billion nationally in 5 years. We estimate this to translate to about $250,000 for southern Doña Ana County.
- A 1% reduction in weight, blood pressure, glucose, and cholesterol risk factors would save $83 to $103 per person annually in medical costs.
- Increasing use of preventive services, including tobacco cessation screening, alcohol abuse screening and aspirin use, to 90 percent of the recommended levels could save $3.7 billion annually nationwide in medical costs or about $350,000 in southern Doña Ana County.
- Indirect costs to employers of employee poor health—lower productivity, higher rates of disability, higher rates of injury, and more workers’ compensation claims—can be two to three times the costs of direct medical expenses.
- Asthma, high blood pressure, smoking, and obesity each reduce annual productivity by between $200 and $440 per person.
- Workers with diabetes average two more work days absent per year.
- Absenteeism costs are reduced by approximately $2.73 for every dollar spent on workplace wellness programs, according to a recent study.

#### 3.5.4. Commuting Time and the Benefits of Public Transportation

In Doña Ana County, the average commuting time in 2015 was 23 min [19]. An estimated personal cost of using a personal vehicle vs. the SCRTD system was calculated using the starting point of Anthony, NM transfer station and a final destination of the Mesilla Valley Intermodal Transfer Station in Las Cruces. This scenario was broken down into two sub scenarios by mode of transportation, private car vs. public transportation. The amount of time spent utilizing public transportation services was multiplied by the minimum hourly wage ($7.50/h or $0.125/min.) of the county in order to place a monetary value on time spent on travelling. Commuting 27.9 m would take approximately 30 min assuming good traffic flow, and on SCRTD service the same distance is travelled in 55 min. The value from the U.S. General Services Administration (GSA) reimbursement rate for personal vehicle travel ($0.54/m in 2016) was multiplied by the number of miles travelled. The U.S. GSA rate accounts for maintenance, insurance, and gas prices to operate a personal vehicle. An estimated cost of a person commuting to work was also calculated considering the counties average commuting time and using same factors. Adjusted for each scenario, costs associated with service, commuting time (on bus and walking), and bus fares were calculated (see Table 6 and Table 7). The costs were calculated based on current bus fares, July 2016. In Table 7, the commuting costs associated with the bus service include the time to travel from home to the bus stop. If the travel time from home to the bus stop were another 30 min each way, this would add another $7.50 to the cost of using the bus service for a round trip (our survey shows that it is somewhat less, averaging about 20 min each way).

In summary, this analysis shows a substantial savings to the traveler by using the bus service compared to a private car, even when considering the extra time required for travelling by bus and accounting for the travel time from home to the bus stop. This should provide an incentive for residents of southern Doña Ana County to use public transportation.

#### 3.5.5. The Costs of Public Transportation

The operating budget for the SCRTD to provide the public transportation services year round in southern Doña Ana County is currently $735,714 per year. The most recent ridership numbers show 206 riders per week. This amounts to a cost per rider of about $69 ($735,714/52 weeks/206 riders). The cost for a single round trip ride from Vado (about midpoint between El Paso and Las Cruces, about 20 m) using Uber ranges from $38–51. Thus, at the current ridership levels the public transportation system is not cost effective compared to other commercial rates for transportation. To make public transportation competitive with commercial rates (about $45/rider), the ridership would need to go up by about 50% on a weekly basis (a total of about 300 riders per week).

## 4. Discussion

### 4.1. Why Desire to Use and Actual Usage Differ

Large differences in consumer preferences assessed through surveys and actual consumer choices are common. With respect to public transportation, in general in the U.S., there is insufficient research to understand and predict the relationship of “desire or willingness to use” and actual usage of public transportation. A few studies conducted outside the U.S. suggest that high quality service and strong marketing campaigns are the most important factors in predicting actual usage of public transportation for new and continuing users [20,21,22,23,24,25].

### 4.2. Recommendations and Predicted Outcomes

Based on the findings of our assessment, we identified the following recommendations.

1. Based on the preponderance of residents in rural Doña Ana County that are in need of public transportation and who said that they would use public transportation if it were available, the SCRTD should implement the bus system for rural Doña Ana County.
2. In order to maintain the operations of the bus system, SCRTD should seek additional funding, including federal and state grants, local government funding, private funding, and any other source that might be available, while at the same time, look for ways to make the bus service more cost effective without reducing quality of the services.
3. Rural residents cannot assume that urban residents will automatically bear all the costs. Recommendations for future HIA practitioners to consider when analyzing public transportation proposals include asking prospective users about: (a) their willingness to pay for some of the costs through fares and how much; and (b) how the costs of the transit system could/should be met.
4. The routes should include stops at Doña Ana Community College campuses, clinics, hospitals, La Semilla Food Center, the Women’s Intercultural Center, and senior centers, as these were the most common preferred destinations.
5. Schedules need to be extended into the early evenings, as people need to board a bus to return home at or near 5:00 p.m. There also needs to be service on Saturdays.
6. SCRTD could also consider other ways to meet rural transportation needs other than through bus service. Other models for rural transit systems include: (a) non-fixed routes based on demand-response; (b) van pools; (c) commuter buses; and (d) demand-response taxis [26].
7. SCRTD needs to develop and implement a communications plan that would include: (a) education of the potential users on how to access and use the bus system; (b) education of the taxpayers about the benefits of the bus system; (c) development of an “identity” that would improve awareness of the system, which could include a clever motto or slogan, brightly painted buses, a “mascot”, improved logo, etc.; (d) improving the visibility and conditions at bus stops to include better signage, advertising, benches, and shade; and (e) marketing the bus system using flyers, posters, and mailings, especially at NMSU (for young people).
8. SCRTD and the county should improve walking conditions around bus stops to include more pedestrian and biking paths to and around the bus stops.
9. Consider buses that use natural gas for fuel; emissions are much reduced compared to gasoline or diesel.
10. More paved roads are necessary for the bus system to fully serve the communities, to provide safer travel of residents to the bus system and to reduce air borne dust in the rural communities. This is the responsibility of the county.
11. Develop an evaluation plan that will document the ridership and overall customer satisfaction with the bus system. This would help maintain public support for the service.
12. Document the benefits of the bus system by collecting data related to health, educational, and economic outcomes, using important indicators that have already been identified for public transportation systems by agencies such as the U.S. Department of Transportation, the U.S. Centers for Disease Control and Prevention, and other agencies. This information could be used to justify the public funding of the bus system.

### 4.3. Lessons Learned

Public transportation in rural areas has the potential to remove a physical barrier that prevents residents from accessing goods and services that are available in urban areas. In particular, limited access to health services in rural areas might contribute to health disparities. Rural residents expressed an overwhelming desire to have access to public transportation (at least 80% of those surveyed). However, during the pilot run of the bus system, few (about 150 riders/week) chose to ride the bus. Ridership has slowly increased to about 500 riders/week in April 2017, but still is far less than our survey would predict. A limitation of our methodology is that the results of our survey did not predict the actual ridership very well, at least on a preliminary time basis. One factor contributing to the limited ridership is an undeveloped “culture” of public transportation use in the region. In other regions of the U.S. a culture of public transportation use has developed that does not exist in the desert Southwest.

Public transportation in rural areas faces several logistical challenges. Chief among them is the difficulty of rural transit systems to efficiently serve multiple pickup points that are some distance apart (several miles) and multiple primary destinations, also several miles apart (as much as 40 m), which a private car or an on-demand service could do more efficiently. There are also challenges associated with matching scheduling to desired destinations at the appropriate time. For commuting to jobs at regular hours, a few transit trips at rush hour might be sufficient, but for other uses, such as meeting medical appointments, more frequent service throughout the day is needed. In this case study, SCRTD struggles to find a middle ground where their service allows users to commute to work and school, but also allows for users to meet doctor’s appointments or shopping without making waiting times too long. SCRTD has considered offering a paratransit service for health care related access, but again these types of services in rural settings have challenges in scheduling and traveling long distances. There are also challenges for customers to get from their home to a bus stop. Rural areas often do not have infrastructure like paved sidewalks for safe walking or bike paths or even paved streets. Buses can accommodate bikes, and many young people do use bikes to get to the bus stop, but many rural customers are elderly and unable to use a bike.

Ultimately, choices regarding rural bus service are political decisions and primarily driven by costs and willingness of taxpayers to “foot the bill”. An issue that we identified is that there is a “structural bias” in urban areas with regards to rural areas. Urban residents ask “why should we bear the cost of providing services to rural areas? Rural residents cannot expect the same services as urban residents.” Urban residents who bear a disproportionate cost of a public transportation system for rural areas feel that if rural residents want the same services as urban residents, they should live in urban areas. Urban residents in general have a stronger political voice due primarily to their larger numbers. However, science-based information and data can help provide validation of rural residents’ concerns and give “voice” to marginalized rural residents. The civic discourse at the time of the original vote on the referendum to raise the general revenue tax to support the bus system was primarily focused on the high cost of the bus system with little or no attention to the benefits. Our HIA gave proponents of the bus system information regarding benefits to counter the conversations dominated by cost and higher taxes. At the time of this writing, the pilot program is still operational, but no decision on permanent funding has been made to support the bus system.

Finally, conducting HIAs in rural areas reveals the complexity of rural areas compared to urban areas, including the political context of local control by locally powerful individuals and how it changes over time. Just as there is structural bias among urban residents against rural areas, there is in rural areas competition among small communities for control and influence and competition among individuals for power and control that is less “in the open” compared to urban areas. Individual stakeholders have “hidden agendas”. Triangulation among stakeholders and key informants can shed light on some of those hidden agendas.

## 5. Conclusions

### 5.1. Conclusions from the Findings of the Assessment

We conclude that public transportation would have major impacts in rural southern Doña Ana County on: (1) health, through improved access to health services and fresh fruits and vegetables; (2) education, through improved access to community colleges, university, and adult learning opportunities; and (3) economic development, through better access to jobs and job training and goods and services. Over 80% of residents responded that they would use public transportation to help improve their health, education, and/or economic status. Priority purposes for accessing public transportation included: doctor appointments, obtaining pharmaceuticals, regular medical treatments, shopping at supermarkets or farmers markets, attending college, visiting a public library, getting a job, attending job training, and paying bills. Preferred destinations included Las Cruces, Anthony, and Sunland Park. An important result was that young people strongly desired public transportation and could benefit significantly from improved access to higher education.

Predicted impacts include: (1) improved health, especially for seniors; (2) improved education, especially for young adults; and (3) improved economic status, especially for families, due to better jobs and better access to goods and services. A major negative impact is the cost of the bus system to the taxpayers. We identified a number of recommendations (Section 4.2), chief of which is that the SCRTD should implement and maintain the bus system for rural Doña Ana County.

### 5.2. Conclusions from the Process of the Assessment

Designing, implementing, and funding public transportation systems in rural areas is challenging. By definition, they serve a relatively small population over a large geographic area. Though it seems that rural citizens should have access to public transportation similar to urban citizens, the expense is not shared proportionately over the population and must be principally borne by urban residents, if financed through tax revenues. This is because there are many more urban residents compared to rural ones. In the case of southern Doña Ana County, the benefits would be significant to an underserved, disproportionately impoverished, racial/ethnic minority community. Such communities as those prevalent in southern Doña Ana County are lacking in resources, economic opportunity, and political voice. These observations and conclusions raise questions of inequity and how inequities relate to, or are even exacerbated by, policies of exclusion in poor rural areas.

We highlight the challenges associated with public transportation in rural areas where there are: (1) long distances to travel; (2) little communication and engagement among communities; and (3) poor road and walking conditions for bus stops. The results of this case study are applicable to low income and fairly disconnected rural areas, where access to health, education, and economic opportunities are limited. There are many similar areas in the southwestern U.S., especially in the border region, other parts of North America, and in developing countries worldwide.

## Figures and Tables

**Table 1:** Demographics of survey respondents.

Characteristic	Number of Individuals Responding	Percent of Total Responding	Doña Ana County Statistics [15]
**Residence**			Population
Chaparral			16,252
Anthony	296	28	9788
Montana Vista	222	21	8592
La Union	116	11	3029
Berino	74	7	4158
Sunland Park	63	6	16,822
Other	53	5	36,190
15 other communities; not more than 20 from any one	232	22	DA County total: 209,233
**Age**			NA
Adolescents (15–25 years)	327	31	
Adults (26–59 years)	433	41	
Seniors (>59 years)	232	22	
Median age = 40 years			
**Gender**			NA
Male	517	49	
Female	539	51	
**Preferred Language**			NA
English	591	56	
Spanish	465	44	
Veterans	63	6	NA
Physically impaired	137	13	NA
Household income <$20,000/year	686	65	Median Income: $38,426

NA: Not Available.

**Table 2:** The top five reasons for using the bus service for each general category of use.

Health	Education	Economics
Medical appointments	Doña Ana Community College	Pay bills
Obtain medications	Public library	Get a better job
Shop at a supermarket for fresh fruits and vegetables	NMSU	Shop at better stores
Regular medical treatment	Museum	Attend community meetings
Shop at a farmers market	GED	Attend a job fair

**Table 3:** Rider characteristics from bus surveys.

Participant Characteristics	Total Sample Size, *n* = 33
**Gender**	
Male	67% (*n* = 22)
Female	33% (*n* = 11)
**Age**	
18–24	12% (*n* = 4)
25–44	24% (*n* = 8)
45–64	55% (*n* = 18)
65+ and over	9% (*n* = 3)
**Primary Transport**	
Bus	55% (*n* = 18)
Personal Vehicle	12% (*n* = 4)
Bicycle	12% (*n* = 4)
Carpool/Rideshare	6% (*n* = 2)
Other	15% (*n* = 5)
**Trip Frequency**	
5 days a week	24% (*n* = 8)
4 days a week	9% (*n* = 3)
3 days a week	9% (*n* = 3)
2 days a week	21% (*n* = 7)
1 days a week	9% (*n* = 3)
23 times per month	6% (*n* = 2)
Once per month	3% (*n* = 1)
First time passenger	18% (*n* = 6)

**Table 4:** Themes for impacts of public transportation identified by key informants.

Economic	Education	Health
Job access and training	Formal education	Access to health services
Access to goods and services	Non-formal education	Fresh fruits and vegetables
Economic rural development	Self-learning opportunities	Promote physical activity
Reducing household costs	Communications and engagement	Reducing risky behaviors among young people

**Table 5:** Prevalence of selected chronic diseases in study area and related absenteeism from work (from 2010).

Disease	Percent Treated from Total Population of NM	Number of Treated Cases in Study Area	Missed Person-Work Days ^1^
Asthma	4.7%	1410	1658
Cancer	4.2%	1260	323
Cardiovascular			
Congestive Heart Failure	0.5%	150	88
Coronary Heart Disease	3.5%	1050	1175
Hypertension	15.7%	4710	1912
Stroke	1.3%	390	1590
Other Heart Diseases	2.1%	630	2712
Depression	6.1%	1800	3320
Diabetes	6.6%	1980	1857
Emergency Department Visits	N/A (70,099 for DA County)	10,515	N/A

^1^ (number of cases) × (% employment among cases/100) × (number of days missed per case).

**Table 6:** Commuting cost to the traveller associated with travelling by personal vehicle.

Commuting	Miles Travelled	Total Costs *
One-way	27.9	$18.82
Round trip	55.8	$37.63
Doña Ana County Average (one-way)	23.0	$15.30

* No costs associated with parking was applied.

**Table 7:** Commuting cost associated with travelling by SCRTD service.

Commuting	Miles Travelled	Total Costs
One-way	27.9 plus half hour to the bus stop	$15.38 (Discounted $14.88) *
Round trip	55.8 plus one hour to and from the bus stop	$30.75 (Discounted $30.25) *

* Reflects discounted fare for seniors and students. The regular fare is $1.00 and the discounted fare is $0.50.
